# Applying the Haddon Matrix to Frontline Care Preparedness and Response in Asymmetric Warfare

**DOI:** 10.1017/S1049023X22001066

**Published:** 2022-10

**Authors:** Flavio Salio, Alessandro Pirisi, Gregory R. Ciottone, Lina Maria Echeverri, Kobi Peleg, Anthony D. Redmond, Eric S. Weinstein, Ives Hubloue, Francesco Della Corte, Luca Ragazzoni

**Affiliations:** 1. World Health Organization (WHO), Geneva, Switzerland; 2. CRIMEDIM, Center for Research and Training in Disaster Medicine, Humanitarian Aid, and Global Health, Università del Piemonte Orientale, Novara, Italy; 3. Beth Israel Deaconess Medical Center, Harvard Medical School, Boston, Massachusetts USA; 4. Department of Emergency and Disaster Management, Tel Aviv University, Tel Aviv, Israel; 5. Humanitarian and Conflict Response Institute, University of Manchester, Manchester, UK; 6. Research Group on Emergency and Disaster Medicine, Vrije Universiteit Brussel, Brussels, Belgium

**Keywords:** asymmetric warfare, emergency and trauma care, Haddon Matrix, Trauma Stabilization Points

## Abstract

**Introduction:**

Asymmetric warfare and the reaction to its threats have implications in the way far-forward medical assistance is provided in such settings. Investments in far-forward emergency resuscitation and stabilization can contribute to saving lives and increase the resilience of health systems. Thus, it is proposed to extend the use of the Haddon Matrix to determine a set of strategies to better understand and prioritize activities to prepare for and set-up frontline care in the form of Trauma Stabilization Points (TSPs).

**Methods:**

An expert consensus methodology was used to achieve the research aim. A small subject matter experts’ group was convened to create and validate the content of the Haddon Matrix.

**Results:**

The result of the expert group consultations presented an overview of TSP Preparedness and Operational Readiness activities within a Haddon Matrix framework. Main strategies to be adopted within the cycle from pre- to post-event had been identified and presented considering the identified opportunities in the context of the possibility of implementation. Of particular importance was the revision of a curriculum that fits the civilian medical system and facilitates its adaptation to the context and available resources.

**Conclusion:**

The new framework to enhance frontline care preparedness and response using the Haddon Matrix facilitated the identification of a set of strategies to support frontline health care workers in a more efficient manner. Since the existing approach and tools are insufficient for modern warfare, additional research is needed.

## Introduction

Asymmetry in warfare is not a new phenomenon. However, in the post-9/11 era, the asymmetry between state and non-state actors and the reaction to asymmetric threats have implications on the provision of medical assistance, in particular trauma care, in conflict zones. The assumption of reciprocity as an ethical imperative and motivation for respecting the law is often unrealistic. Instead, strategies to cause greater loss of human life represent a crude reality. These include the use of prohibited modalities and selection of civilian targets to replace military ones, posing major pragmatic and ethical challenges to prepare for and respond to the needs of the affected population.^1^

Military research focusing on the understanding of overall severity of injuries and other non-medical factors contributing to survival and long-term recovery has resulted in improvement in the clinical outcomes of injured soldiers throughout the battlefield trauma system.^2^ Reducing the time from point-of-injury (POI) to arrival at a medical facility dramatically decreased death rates of battlefield casualties. At the same time, this has created a complex system of levels of care, previously referred to as echelons, based on differences in capability and not quality of care.^3^

Attempts to define and evaluate the application of a similar model involving civilians, recognizing the significant shift in the provision of medical care in such contexts from humanitarian organizations, have been registered. Wars in recent decades have been characterized by an increasing number of civilian casualties. Civilians will usually lack extra body protection used for example by the military, and this is reflected in their pattern and severity of injury. Although comparative analyses between systems should be performed, situations of armed conflict or other emergencies in unsecure environments are extremely context-specific and require constant analysis and adjustment based on realities on the ground and tactical circumstances.^2,4^

Rapid evacuation from the POI and establishing far-forward emergency resuscitation and stabilization capabilities are both needed to save lives and reduce disabilities.^5^ However, limitations derived from the lack of a prehospital trauma care system exist in many low- and middle-income countries and are associated with the growing number of hybrid threats. Increasing the availability of medical transport and trained health care providers, as well as preparing for the most likely scenarios with contingency plans in the event conditions deteriorate, should be pursued.^6^

Recognizing the challenges of moving life-saving interventions closer to the POI, and the implications to civilians of today’s asymmetrical warfare, it can be argued that the initial part of the trauma care system is being neglected.^7^ Investments in far-forward emergency resuscitation and stabilization can contribute to saving lives and increase the resilience of health systems. Efforts to improve preparedness measures and system competencies should be prioritized, utilizing different scenarios that ensure accountability and prompt actions.

Although the Trauma Stabilization Point (TSP) has already been described as the first site of care staffed by trained medical personnel, further research is required to better define its scope and operationalization.^8^ In line with this, the authors propose the use of the Haddon Matrix, which has been used for more than two decades by injury prevention professionals, to evaluate contributing factors, design response strategies, and promote safety.

The matrix provides a conceptual framework that helps to examine problems systematically, breaking them down into smaller components to propose actions, proving to be an effective planning tool. It can help health leaders and planners in their decision-making process, analysis of threats and risk factors, identification of priority actions, allocation of resources, and after-action review.^9^ Therefore, the benefits of its application and use have been extended beyond injury prevention to better understand different public health issues and support public health emergency preparedness.

Hence, the aim of this study is to propose the use of the Haddon Matrix to determine a set of strategies to better understand and prioritize activities to prepare for and set-up frontline care in the form of TSPs.

## Methods

### Study Design

An expert consensus methodology was used to achieve the research aim. This includes a qualitative research method and data collection technique in the form of focused group discussion. To obtain a thorough understanding on the current trend of modern armed conflicts, a search was conducted on the Global Terrorism Database (GTD), the Armed Conflict Location and Event Data Project (ACLED), and Uppsala Conflict Data Program (UCDP). Additionally, review of the existing documentation and data from the implementation of the TSP in the context of the Mosul’s battlefield (Iraq) was performed.

In order to gain an understanding of relevant technical and operational considerations related to the TSP, a small subject matter experts’ group was convened. It was tasked with: (1) the review and discussion related to the problem, its magnitude, and the agreement on the need of such intervention; (2) the creation of a list of main actions to perform before, during, and after the implementation of the TSP; and (3) the validation of the content of the Haddon Matrix.

The recorded discussions and notes were then transcribed. Two rounds of discussion occurred aimed at narrowing down an initial list into tangible activities. This iterative process continued until the conversations reached saturation and consensus was obtained.

### The Haddon Matrix

The Haddon Matrix is comprised of three rows representing the phases of an injury, namely pre-event, event, and post-event, and four columns representing the contributing and influencing factors (host, agent/vehicle, physical environment, and social environment). The host column refers to the person at risk of injury. The agent refers to the energy that is transmitted to the host through a vehicle or vector. Physical environment refers to the characteristics of the setting where the event takes place. Social environment refers to the law and social norms associated with the location of the event. The terminology used for the factors of the matrix can be modified based on the context of its application.^10^

Considering the majority of the unwanted events occur sequentially or in phases, each row presents opportunity for prevention or control. The identification of contributing and influencing factors guide the definition of strategies to be adopted in each phase.

### Expert Group

The criteria used to guide the selection of the expert group considers individuals representing different disciplines essential to the successful creation of the Haddon Matrix. Highly trained and competent within their specialized area of knowledge and expertise, the ten experts out of the twelve initially invited have expertise and experience in trauma and emergency care, humanitarian operations, military interventions, policy, and conflict analysis (Table 1).

**Table 1.** Expert Panel Demographics and Basis of Expertise

Expert Panel	
**Gender**	**N**
Male	8
Female	2
**Country of Residence/Work**	**N**
United States	2
United Kingdom	2
Norway	1
Israel	1
Switzerland	1
Italy	1
Colombia	1
Ukraine	1
**Current Role**	**N**
Professor	3
Physician	1
Trauma Nurse	1
Medical Director	2
Director of Operations	2
Health Cluster Coordinator	1
**Years Working in the Field**	**N**
20+ years	4
15+ years	5
10+ years	1
**Expertise**	**N/10**
Trauma and Emergency Care	7
Humanitarian Operations	7
Military Interventions	2
Health Policy	3
Conflict Analysis	1

Invitation to contribute to the expert group was circulated by email to all ten members, including a brief document with the explanation of the objective of the study and instructions for participation. Although the Haddon Matrix model wasn’t known to all of them, the advantages of the expert panel composition were the knowledge and experience in conflict setting, as well as familiarity with the TSP concept.

During the first virtual meeting, the moderator introduced the Haddon Matrix, the findings of the search, the purpose of the study, and background on the TSP. Based on the brainstorming method, the list of contributing and influencing factors was created on the basis of three main aspects: medical response staff, frontline care requirements, and characteristics of asymmetric warfare that affects the response in relation to the three phases (pre-event, event, and post-event). The event phase spanned the activation and set-up of the TSP through to its deactivation and/or relocation due to possible changing in the pattern of presentations, conflict dynamic, and intensity, which can provoke significant population movement.^4,11^

The list of contributing and influencing factors was reviewed, items merged, and recorded in the Haddon Matrix. Final confirmation from the expert group was received at the end of the second virtual meeting and by email from all the experts.

### Ethics Approval and Consent to Participate

This study obtained approval from the Cross-Corporate Ethics Committee of Novara (Comitato Etico Interaziendale di Novara) on March 3, 2020 (protocol ID: 2/20). All participants granted their informed consent for the use of the information they provided.

## Results

**Figure 1.**
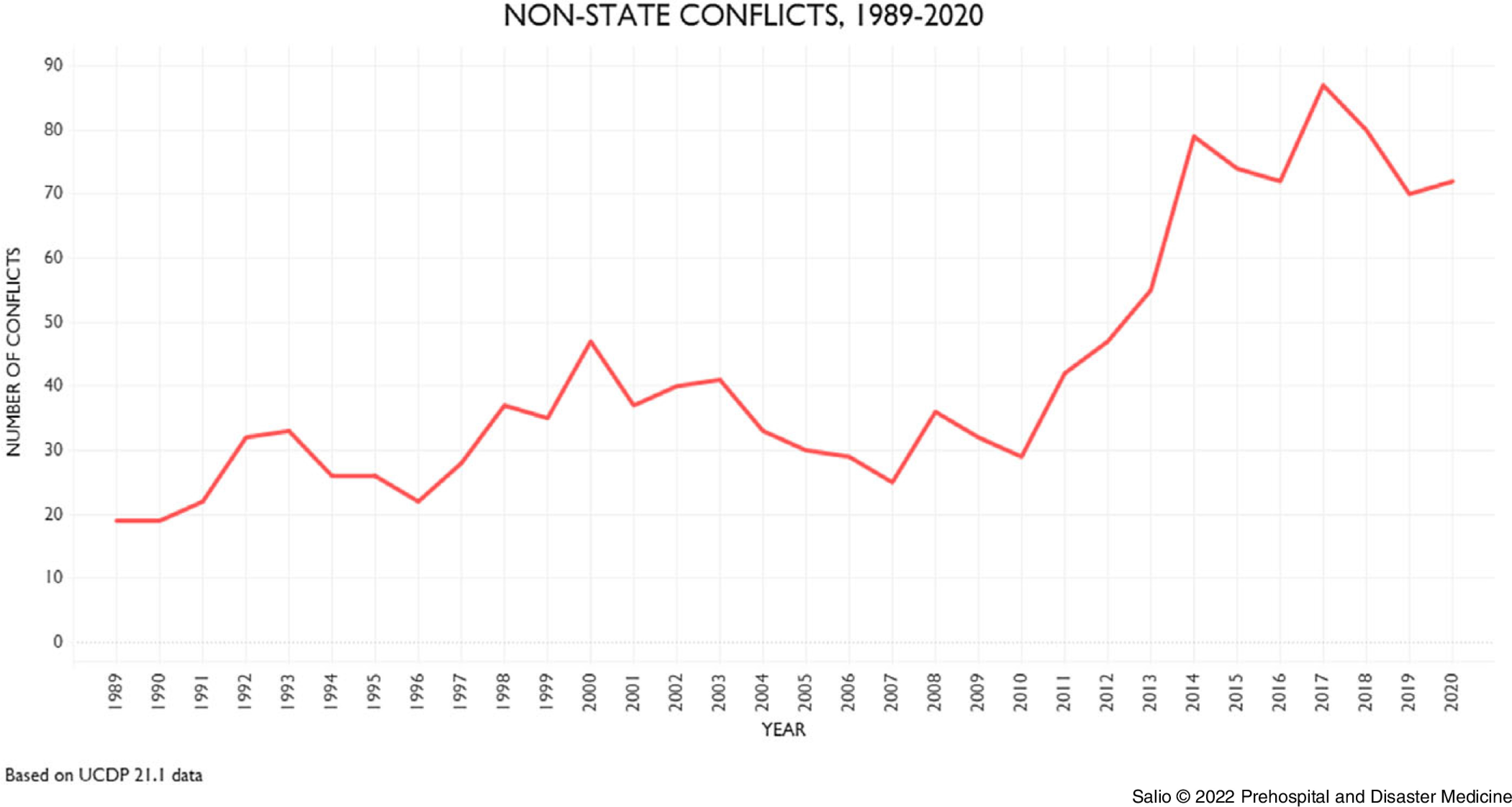
Non-State Conflicts, 1989-2020.

**Figure 2.**
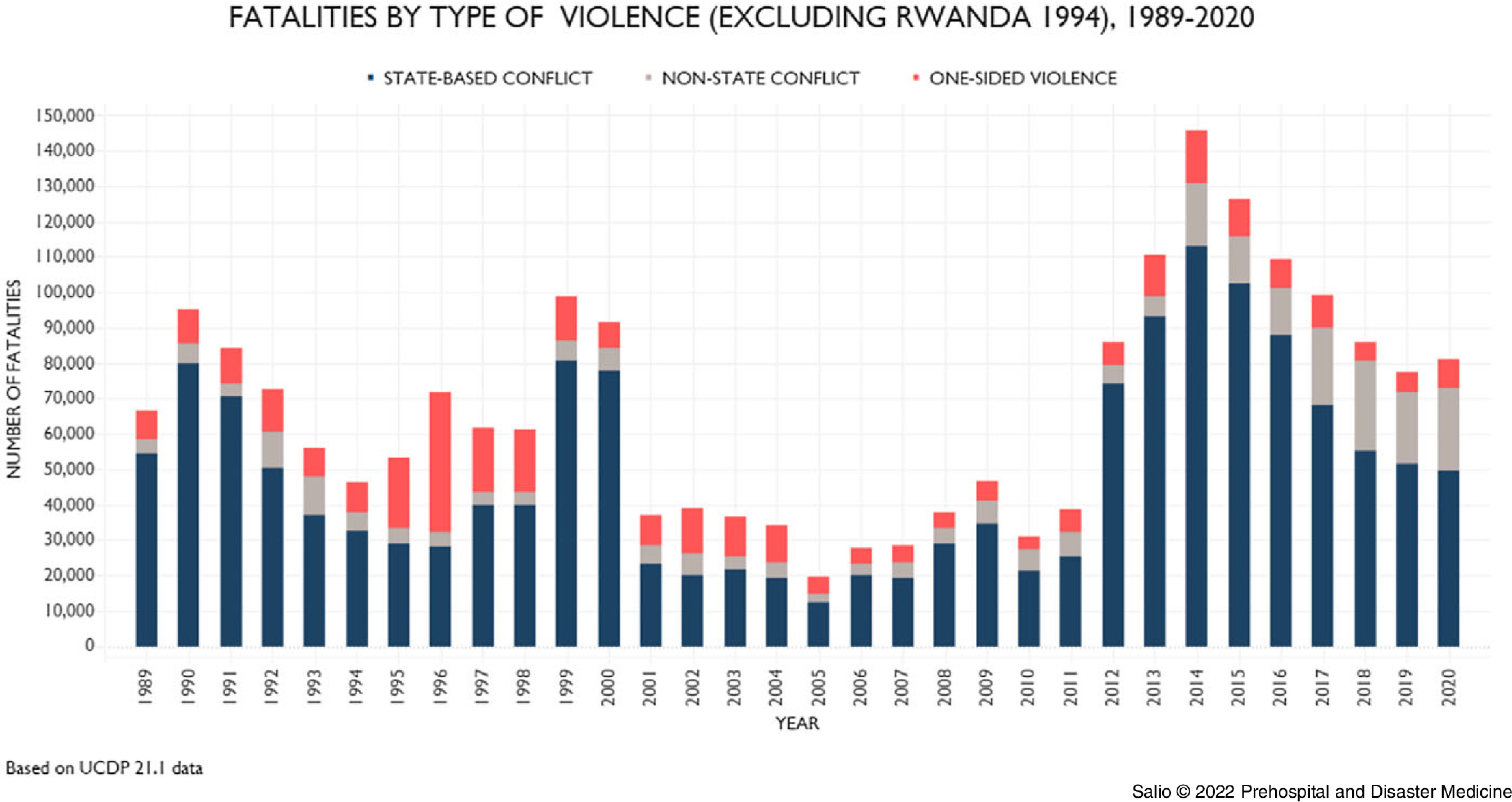
Fatalities by Type of Violence, 1989-2020.

The review of the trend of modern armed conflicts demonstrated a significant increase in non-state conflicts (Figure 1) and fatalities (Figure 2) in the last decade. Modern conflicts take civilians lives, and increasingly, incidents of attacks on health are reported and documented.^12^ However, estimating and reporting the number of civilian casualties is increasingly challenged by organizational, political, strategic, and tactical hurdles.^7,13^

**Figure 3.**
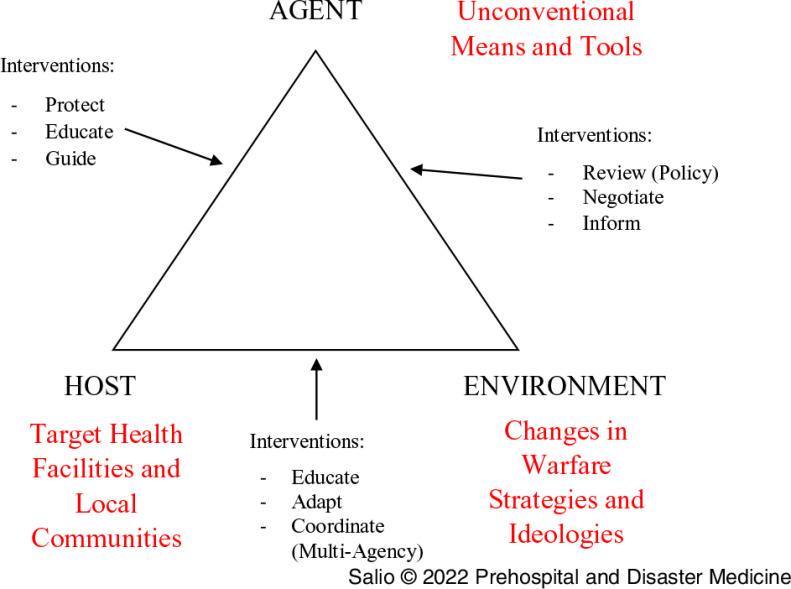
Epidemiological Triangle.

The outcome of the initial review and first task assigned to the expert group are reflected in the epidemiological triangle (Figure 3). It shows the correlation between each of the factors in conjunction with macro level characteristics associated with asymmetric warfare and interventions required to prevent or mitigate the effects of war-related actions.

### Expert Group - The Haddon Matrix

A list of 148 elements was initially created to cover the cycle from pre- to post-event. Elements were subsequently categorized based on similar patterns or characteristics and assigned to their related boxes within the matrix.

The first phase included elements that need to be contemplated prior to the event’s occurrence, the preparedness, and level of readiness required to maximize a speedy and effective response. The event’s phase considered those factors and actions that should be taken in order to minimize impact and adverse outcome. The final phase included actions that should be carried out to minimize mortality and morbidity related to the event. This included the variation of intensity and possible multiple relocations of the TSP.

Table 2 presents the result of the expert group consultations, which describes the overview of TSP Preparedness and Operational Readiness within a Haddon Matrix framework. A number (ten) of opportunities or potentially modifiable factors had been highlighted as priorities due to their possible positive impact on the successful implementation of the TSP.

**Table 2.** Haddon Matrix and TSP Preparedness and Operational Readiness

	Contributing and Influencing Factors			
Phases	Host (Human Factors)	Vector and Vehicle	Physical Environment (Overall Design)	Social Environment (Social and Cultural Norms, Policies)
Pre-Event	Training to increase willingness and ability to respond* Training to increase knowledge and familiarity with hazard* Exercise on eligibility criteria and activation* HR selection criteria	Type of agent (human, physical, mechanical, thermal, chemical, biological, radiation) Medical intelligence including the risk analysis*	Pre-event testing of temporary infrastructure and equipment* Scene assessment Supply stockpile*	Guidelines for frontline workers and local communities* Intra- and inter-institutional relations Budget (preparedness resource allocation)
Event	Command structure HR management including remote management system Speed of activation and accountability of HCW* Patient tracking, record, and discharge plan*	Structural failure Protective devices Decontamination capability	Site selection, design including holding area Water, sanitation, and energy Resupply and cold chain	Public information sharing policy Re-assess variables HCW including ambulance access to the area
Post-Event	Comfort in role flexibility* Psychological support and long-term follow up Confidentiality Management of patient records	Secondary effects	Resupply Waste management	Revision of the scope of the mission After action report Community-based resilience

Abbreviations: HCW, health care worker; HR, human resources; TSP, Trauma Stabilization Point.

* Opportunities–potentially modifiable factors.

This was the result of a broader agreement within the expert group on the content of the matrix. Additionally, it proved to be useful as an analytical framework in support of the identification of main activities to be prioritized for the implementation of frontline care in the context of asymmetric warfare.

The richness of the discussion, and the knowledge and expertise of the expert panel, had facilitated the analysis of the outputs of the Haddon Matrix. This, in conjunction with the number of comments provided during the sessions and through email exchanges, inspired the use of the reverse matrix approach. Table 3 presents the application of this approach to the pre-event phase, and more precisely, the activities and procedures necessary for the preparation and activation of the TSP. As per previous attempts, it revealed gaps in knowledge and evidence providing possible areas for future research.^14^ Its use should be carefully analyzed and evaluated further.

**Table 3.** Reverse Haddon Matrix (Pre-Event - Activation of the TSP)

	Contributing and Influencing Factors			
Phases	Host (Human Factors)	Vector and Vehicle	Physical Environment (Overall Design)	Social Environment (Social and Cultural Norms, Policies)
Pre-Event (Activation of the TSP)	To what extend training increases willingness to respond? Ethical considerations for engaging in frontline trauma care? To what extent risks for frontline workers can be mitigated? Does previous military experience increase the confidence of TSP personnel?	Does medical intelligence reduce risk of exposure? How to prioritize/tailor CBRN-e and HAZMAT knowledge and training for specific risks?	Which standards should be applied for the definition of the structure and equipment needed? Which is an effective and efficient stockpile strategy?	Which are the barriers to prepare for and to train frontline workers? Are current policies adequate for the provision of care in modern warfare? Which are the barriers to engage and maintain effective civil-military cooperation? Cost effectiveness of such intervention?

Abbreviations: CBRN-e, chemical, biological, radiological, nuclear, and explosives threats; HAZMAT, hazardous material; TSP, Trauma Stabilization Point.

## Discussion

Using an expert consensus methodology, this study describes the creation of a new framework to enhance frontline care preparedness and response in asymmetric warfare. By applying the Haddon Matrix, this framework will facilitate the understanding of the main strategies to adopt and the key activities to perform before, during, and after the implementation of TSPs in conflict settings. The model allows its users to better understand the multi-dimensional nature and interdisciplinary perspectives of this form of medical intervention. This includes disciplines such as engineering, law, medical, and behavioral sciences to assist in the preparedness, operational readiness, and response of such far-forward medical capability. In the following discussion, main strategies are elaborated that consider the identified opportunities in the context of the possibility of implementation.

### Pre-Event

*Training and Drills*—Significant importance is being given to training and drills to increase confidence and the willingness to respond among health professionals, along with familiarity of relevant hazards. However, current existing guidelines on Tactical Combat Casualty Care (TCCC) do require adaptation to the scope of practice and the needs of the civilian medical and operational environments. Emphasis remains on the ability to provide far-forward emergency resuscitation and stabilization in remote and resource-limited settings.^15^ The risk of a broader (and inappropriate) spectrum of procedures performed at the site should be considered together with its scope. For example, evidences suggest the need for more research on the impact of hemorrhage control training for first responders on patient outcomes.^16^ Another example being debated in the literature and contested is the far-forward provision of whole blood, especially when facing transport times and other logistical constraints.^17^

Increasing attacks targeting civilians, along with the use of non-conventional means and the threats they pose to the health system, suggest the need for new approaches and investments to adequately equip frontline health care providers.^18^ Of particular importance is the revision of a curriculum that fits the civilian medical system and facilitates its adaptation to the context and available resources.

*Medical Intelligence—*For the purpose of this study, medical intelligence is defined as a critical capability to monitor and evaluate risks to health and frontline health care personnel. It aims to mitigate risks, regardless of how low the probability is. This includes threat detection and identification, information on the operational context, and its characteristics. The ability to conduct and analyze information from various risk assessments, a system for monitoring, and quality improvement actions should be included as core elements for medical planning and operations.^19,20^ Clear distinction should be made between military and civilian intelligence systems, including their objectives and roles in conflict settings. To improve the system, additional research is needed to address, among other things: differences between injuries to soldiers on the battlefield and civilians injured where they live; the training and requirements for field management in these two different areas; and incorporating distance from hospitals and nature of injuries.

*Infrastructure and Equipment—*Both infrastructure and equipment should be located near areas where casualties are likely to occur. Principles should include considerations for areas providing passive security, inside buildings or in field settings, with the possibility to expand capacity if casualty load increases. There should be clear access to evacuation routes and evacuation assets. To note, evacuation policy and procedures must be established beforehand and amended as situations evolve, as they may represent a source of friction during the course of operations. The care provided can be impacted by several operational constraints and nonmedical factors. For example, weather and environmental factors and their related heat and cold injuries can be some of the first threats to be encountered. Medical equipment and consumables should be in line with the provider scope of practice and adequate for the procedures that are expected to be performed and number of patients expected.^21^ Properly equipping and training has been suggested as a new approach to enhancing the military medical system.^22^ Recognizing differences in the delivery of trauma care in the military and civilian sectors, as well as some similarities in the management of trauma patients, it is fair to assume that the adoption of this approach could be extended to the civilian frontline medical system.

### Event

*Command Structure and Activation—*When setting up a TSP, referral lines and communication channels among the different levels of care need to be well-identified and disseminated among those who participate in the operation. Uncertainty affects strategic location decisions with possible impact on tactical and operational decisions, and ultimately on patient outcomes. The friction between current operations and the need to improve capabilities need to be balanced by the ability to maintain a perpetual state of team readiness. There is a greater call for more standardization and necessary guidance to support teams deploying into isolated and challenging environments with limited resources and self-reliance to optimize patient survival.^23^ Additional considerations include particular attention on crisis risk communication, the speed of notification, and rotation of personnel.

*Execute and Reassess—*The importance of having trained providers with experience and expertise relevant to their assigned roles, and a wide-range of professionals who directly support the clinical mission, is highlighted in multiple studies.^24^ Optimal trauma care and patient outcomes require provision of life-saving interventions at the POI to increase chance of survival and coordination with other facility-based services. The pattern of presentations includes patients with acute, complex, penetrating polytrauma, and multiple injuries from high-energy transfer fragments, such as ordnance, bullets, and blast wounds. The work environment is characterized by higher workload, hostile surroundings, and limited resources.^25^ Thus, constant reassessment of the variables, or external factors that should be taken into consideration in the set-up of the TSP, should be performed and threat-based interventions prioritized.

### Post-Event

*Comfort in Role Flexibility and Psychological Support—*The importance of comfort in role flexibility should be considered across the three phases. It is defined by the institutional ability to instill workers’ self-efficacy in the roles they will perform and their engagement and value to accomplish the assigned mission.^26^ General recommendations for staff exposed to a potentially traumatic event include the immediate provision of psychological first aid and assessment by a mental health professional, within one to three months from the incident, to determine whether further follow-up care is required. To note, pre-deployment programs and research are on-going to build psychological resilience of military and emergency medical personnel before possible exposure to traumatic events.^27^ Although there isn’t enough evidence to support these efforts, future research is needed. This should be aligned with outcomes from the data related to psychological evaluation and services provided to staff post-deployment. Additionally, it is important to empower the community and invest in training on the provision of basic care. This can have a potential to increase resilience and save lives, as most of the injured will first be treated by bystanders. The costs will be far less than what modern armed conflict may generate.

*After Action Review and Evaluation—*How does one define mission success from a medical standpoint? The definition of monitoring and quality improvement systems helps the TSP and the entire trauma care system to periodically assess the adequacy of the chain of casualty care, the efficiency of frontline measures, and the evacuation system itself. Civilian and military key performance indicators (KPIs) can be adapted by the health care delivery system that is managing the TSP, including the components and assets involved to meet the specifics of asymmetric warfare trauma care. Relocation of the TSP should follow pre-defined indicators, first and foremost, the safety of the health care providers, patients, and families, as well as these KPIs to maximize the distance between POI and the TSP. The model can be used to verify the effectiveness of the intervention, improve frontline medical response, and advocate for investments in this area. Countries exposed to the risk of conflicts and violence could combine such an approach with efforts to strengthen existing structural limitations derived from the lack of a prehospital trauma care system.^28^

## Limitations

The findings of the study are the result of the experience and opinions of those senior experts that participated in the research. Face-to-face interaction and socialization would have facilitated a greater contribution and collaboration, resulting in faster completion of the study. An analysis of the ethical implications for health care professionals providing such kind of services in austere and non-permissive environment is not being included in the scope of the study.

## Conclusion

Modern warfare has challenged the way in which far-forward medical assistance is provided in such settings. This study presented a new framework to enhance frontline care preparedness and response using the Haddon Matrix. The Haddon Matrix provides a user-friendly way to systematically describe the key factors that affect the delivery of frontline trauma care. As an effective planning tool, it facilitates the identification of strategies to support the preparedness and operational readiness of frontline health care workers in a more efficient manner. Since the existing approach and tools are insufficient for modern warfare, additional research is needed.
